# Plant extract mixture shows anti-inflammatory and barrier-strengthening effects and activates aryl hydrocarbon receptor in a 2D psoriasis model

**DOI:** 10.1038/s41598-026-50000-8

**Published:** 2026-04-28

**Authors:** Nina Heinemann, Franziska Rademacher, Henning Vollert, Regine Gläser, Jürgen Harder

**Affiliations:** 1 Department of Dermatology, Quincke Research Center, Kiel University, Kiel, Germany; 2 Bioactive Food GmbH, Bad Segeberg, Germany; 3 Department of Dermatology Quincke Research Center, Kiel University, Rosalind-Franklin Str. 9, 24105 Kiel, Germany

**Keywords:** Psoriasis, Phytochemicals, Aryl hydrocarbon receptor, Antioxidant, Inflammation, Filaggrin, Biochemistry, Diseases, Drug discovery, Plant sciences

## Abstract

Inflammation, defect skin barrier and oxidative stress contribute to the pathogenesis of psoriasis, a common chronic inflammatory skin disease. The aryl hydrocarbon receptor (AhR) represents a promising treatment target as its activation by specific ligands attenuates inflammatory processes and barrier disruption in the epidermis. Recently, we demonstrated that a plant extract mixture consisting of apple, curly kale and green tea activates the AhR and shows beneficial effects in an atopic dermatitis skin model. The aim of this study is to investigate potential beneficial effects of the plant extract also in psoriasis using an in vitro 2D psoriasis model. Gene expression analysis revealed that the extract restored the expression of the skin barrier molecule filaggrin, reduced the expression of inflammatory markers and inhibited the psoriasis-typical overexpression of antimicrobial peptides. A luciferase reporter assay demonstrated that the extract activated AhR in the 2D psoriasis model. Suppression of AhR via siRNA revealed that the filaggrin induction was mediated by AhR activation, while the anti-inflammatory effects were AhR-independent. Furthermore, the extract exhibited antioxidant activity against intracellular reactive oxygen species. In summary, the anti-inflammatory, antioxidant and barrier-strengthening effects of the plant extract mixture in the 2D psoriasis model suggest that the plant extract may represent a promising topical treatment option in psoriasis.

**Supplementary Information:**

The online version contains supplementary material available at 10.1038/s41598-026-50000-8.

## Introduction

Psoriasis is a common chronic inflammatory skin disease affecting approximately 2% of people worldwide^1^. The most frequent form is psoriasis vulgaris, also known as plaque psoriasis due to its clinical phenotype of sharply demarcated red plaques covered by silvery-gray scales at different skin locations^1^. The etiology behind is complex and multifactorial. A combination of genetic risk factors and environmental triggers is discussed to induce an overactivation of the immune system leading to inflammation and hyperproliferation in skin. The inflammation is mainly driven by the interleukin (IL)-23/T helper cell (Th)17 axis, which describes the complex crosstalk between dendritic cells, Th17 cells and epidermal cells leading to an inflammatory feed-forward loop^1,2^. In brief, dendritic cells secreting IL-23 activate Th17 cells, which then infiltrate the epidermis and secrete cytokines such as IL-17 A. These cytokines activate different signaling pathways such as the Nuclear Factor kappa-light-chain-enhancer of activated B cells (NF-κB), Signal Transducer and Activator of Transcription 3 (STAT3) and Mitogen-Activated Protein Kinase (MAPK) pathways in keratinocytes resulting in a strong overexpression of antimicrobial peptides (e.g. S100 calcium-binding protein A7 (S100A7, psoriasin) and Defensin Beta 4 A (DEFB4A)), induction of several pro-inflammatory genes (e.g. IL-1β, Tumor Necrosis Factor Alpha (TNFα) and IL-36γ) and chemokines such as C-X-C Motif Chemokine Ligand 8 (CXCL8)^3,4^. These chemokines function as chemoattractants for T cells, thereby closing the self-sustaining cycle of inflammation.

The inflammatory environment induces hyperproliferation in keratinocytes and inhibits the expression of differentiation markers such as filaggrin (*FLG)* and loricrin (*LOR*). This dysregulation leads to skin lesions and barrier dysfunction^5^. Furthermore, increased vascularity is typically observed in psoriatic skin caused by abnormal angiogenesis driven by different proangiogenic factors such as the vascular endothelial growth factor A (VEGFA), which is upregulated in skin lesions^6^. The newly formed blood vessels contribute to the pathology of psoriasis by facilitating the migration of inflammatory cells into the skin and promoting hyperproliferation by improved nutrient delivery^7^.

Oxidative stress is another factor implicated in the pathogenesis of psoriasis, as elevated reactive oxygen species (ROS) and dysregulated antioxidative response were observed in serum and skin of psoriasis patients^8^. It has been suggested that oxidative stress itself contributes to psoriasis by enhancing immune activation, promoting keratinocyte hyperproliferation, and driving inflammation through ROS-mediated signaling pathways^9^.

Besides the physical symptoms, such as itching and burning skin, psoriasis patients often suffer from stigma and discrimination, which can lead to psychological disorders such as social anxiety or depression^10^. Efficient and safe treatment options are therefore of high importance for relieving symptoms and improving the quality of life in these patients.

Even though there is no cure today, the improved understanding of the underlying molecular mechanism of the disease led to the development of key pathway-modulating biologic agents, which show high efficacy in the treatment of psoriasis^11^. Nevertheless, systemic therapeutics, such as biologics, are primarily recommended for moderate-to-severe psoriasis, whereas in cases of mild psoriasis, topical treatments, such as corticosteroids, calcineurin inhibitors, and vitamin D analogues, are used in the majority of cases^12,13^. These topical treatments may cause local side effects, especially at sensitive skin areas, and thus novel topical treatments are still needed to better help many psoriasis patients^14^.

Recently approved by the Food and Drug Administration (FDA) of the United States in 2022, tapinarof expanded the spectrum of topical therapeutic options and offers a novel mode of action by targeting the aryl hydrocarbon receptor (AhR)^15^. The AhR is a ligand-dependent transcription factor and can be activated by a variety of endogenous and exogenous ligands, with the resulting downstream effects highly dependent on the specific type of ligand, species and tissue^16^. In skin, AhR plays an important role in barrier function and regulation of inflammation^17^. Accordingly, AhR activation by tapinarof in psoriatic conditions results in the downregulation of inflammatory cytokine expression in skin, restoration of skin barrier molecules such as filaggrin as well as the induction of antioxidative enzymes such as NAD(P)H: quinone Oxidoreductase 1 (NQO1) via the AhR-Nuclear Factor Erythroid 2–Related Factor 2 (Nrf2) pathway^13,18^. Beneficial effects on skin health were also reported for other AhR agonists, which underscores the promising role of AhR as novel therapeutic target for psoriasis treatment^19,20^.

Previously, we have seen that a plant extract mixture consisting of green tea, apple and curly kale extract activates the AhR and exhibits anti-inflammatory, *FLG*-inducing and antioxidative effects in a 2D atopic dermatitis model^21^. These characteristics of the plant extract raise the question of whether it may also exert beneficial effects in psoriasis, where skin barrier defects with decreased *FLG* expression and increased levels of reactive oxygen species are observed too^5,8^. The AhR activating properties of the extract in the AD model suggest potential therapeutic benefits in psoriasis, given that other AhR ligands such as tapinarof exhibits positive effects both in AD and in psoriasis^19^. Thus, this study aimed to investigate whether the plant extract mixture has a positive effect in a 2D psoriasis model, and how the AhR contributes to these effects.

## Results

### Plant extract mixture restores gene expression of skin barrier molecules and lowers inflammation in a 2D psoriasis model

**Fig. 1:**
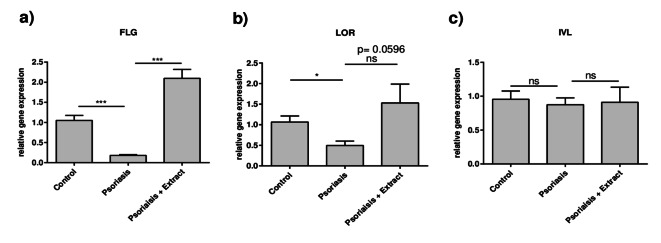
Plant extract mixture restores gene expression of skin barrier molecules filaggrin and loricrin in the 2D psoriasis model. CaCl_2_-differentiated NHEKs were left unstimulated (control) or stimulated with a psoriasis-like cytokine mixture (IL-1β, IL-17A, IL-22 and TNF-alpha, each 10 ng/mL) either in the absence (Psoriasis) or in the presence of the plant extract (Psoriasis + Extract) for 21 h. Gene expression levels of (**a**) *FLG*, (**b**) *LOR* and (**c**) *IVL *were determined by real-time PCR. Statistical significance was tested by a, c) one-way ANOVA with subsequent Sidak’s multiple comparison test or b) Kruskal-Wallis test with subsequent Dunn’s multiple comparisons test (*n* = 12 *p < 0.05; **p < 0.01; ***p < 0.001; ns = not significant).

**Fig. 2:**
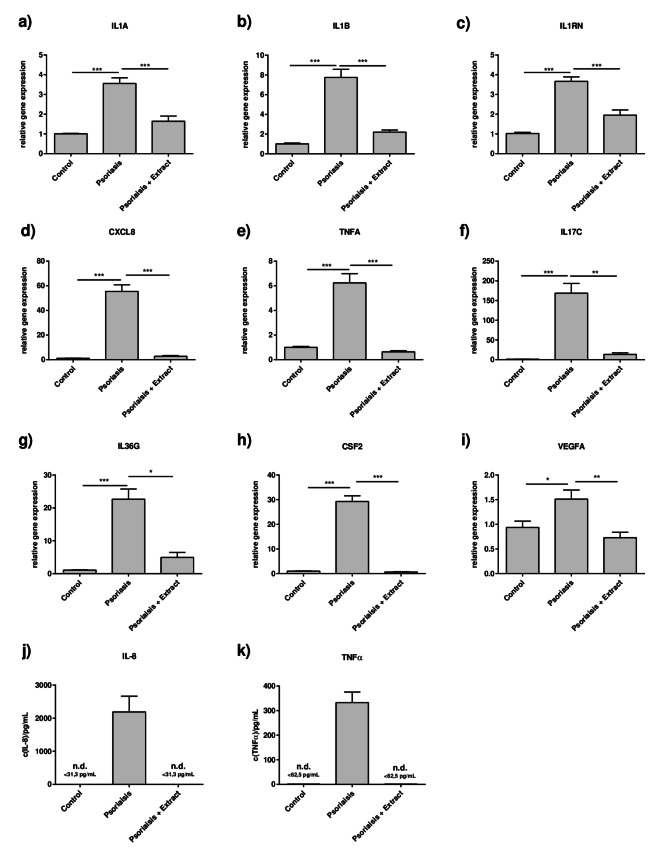
Plant extract mixture downregulates inflammatory markers in the 2D psoriasis model. CaCl_2_-differentiated NHEKs were stimulated as described in Fig. 1. Gene expression levels of (**a**) *IL1A*, (**b**) IL1B, (**c**) *IL1RN*, (**d**) *CXCL8*, (**e**) *TNFA*, (**f**) *IL17C*, (**g**) *IL36G*, (**h**) *CSF2*, (**i**) *VEGFA* were measured. Statistical significance was tested by a, b, c, e, h) one-way ANOVA with subsequent Sidak’s multiple comparison test or d, f, g) Kruskal-Wallis test with subsequent Dunn’s multiple comparisons test (*n* = 12, *p < 0.05; **p < 0.01; ***p < 0.001; ns = not significant). Protein expression levels of (**j**) IL-8 and k) TNFα were measured in the supernatants of the cells by ELISA. Values for unstimulated control and plant extract-treated cells were below the detection limit of 31,3 pg/mL (IL-8) or 62,5 pg/mL (TNFα) and not detectable; therefore, no statistical analysis was performed. n.d.= non-detectable.

To determine if the plant extract has potential beneficial effects on psoriasis, a 2D psoriasis model was utilized in which normal human epidermal keratinocytes (NHEKs) were treated with a psoriasis-like cytokine mixture consisting of IL-17A, TNFα, IL-1β and IL-22 (each 10ng/mL) for 21 h with and without the plant extract mixture. Then, the gene expression levels of psoriasis-relevant genes were analyzed (Fig. 1 + 2). It was shown that the 2D psoriasis model mimicked the psoriasis gene expression signature of barrier molecules well, as the skin barrier molecules *FLG* and *LOR* were downregulated (Fig. 1a + b). Gene expression of the early differentiation marker involucrin (*IVL*) was not altered in the 2D psoriasis model (Fig. 1c). In addition to the downregulated barrier molecules, an increased expression of psoriasis-associated inflammatory markers, such as *IL1A*,* ILB*, *CXCL8*,* TNFA*, *IL17C*, *IL36G* and *CSF2*, was observed (Fig. 2a-h). Furthermore, the expression of the pro-angiogenic factor *VEGFA* was induced in the model, too (Fig. 2i).

After confirming the suitability of the 2D psoriasis model, the effects of the plant extract mixture were analyzed. Stimulation of the 2D psoriasis model with the extract significantly restored the gene expression of the barrier molecule filaggrin (Fig. 1a) and loricrin was elevated by trend (Fig. 1b, *p* = 0,0596). Additionally, the extract exhibited anti-inflammatory effects, as the high gene expression levels of all measured inflammatory mediators in the 2D psoriasis model were suppressed by the extract (Fig. 2a–h). In line with this, the gene expression of *VEGFA* was reduced to control level by the extract (Fig. 2i). Consistent with the gene expression results, enzyme-linked immunosorbent assay (ELISA) confirmed that the extract reduced the strongly induced protein expression of IL-8 and TNFα in the 2D psoriasis model (Fig. 2j + k).

### Plant extract lowers upregulated antimicrobial peptide expression in the 2D psoriasis model

**Fig. 3:**
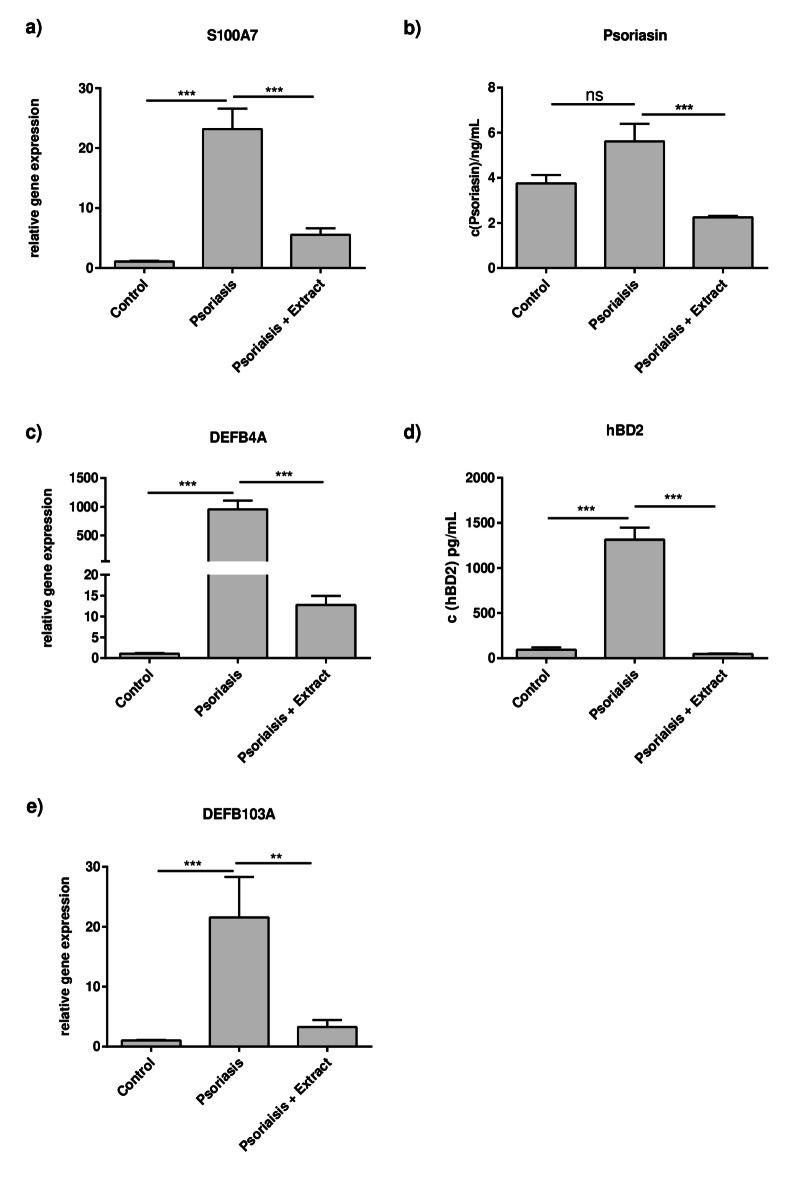
Plant extract mixture lowers upregulated antimicrobial peptide expression in the 2D psoriasis model. CaCl_2_-differentiated NHEKs were stimulated as described in Fig. 1. Gene expression levels of (**a**) *S100A7*, (**c**) *DEFB4A* and (**e**) *DEFB103A *were measured. Statistical significance was tested by (**a**, **c**) one-way ANOVA with subsequent Sidak’s multiple comparison test or (**e**) Kruskal-Wallis test with subsequent Dunn’s multiple comparisons test (*n* = 12). Protein expression levels of (**b**) psoriasin and (**d**) hBD2 were measured in the supernatants of the cells by ELISA. Statistical significance was tested by b) Kruskal-Wallis test with subsequent Dunn’s multiple comparisons test (*n* = 12) or d) one-way ANOVA with subsequent Sidak’s multiple comparison test (*n* = 6, *p < 0.05; **p < 0.01; ***p < 0.001; ns = not significant).

Besides the classical inflammatory mediators, antimicrobial peptides (AMPs) are highly upregulated in psoriatic skin and contribute to inflammation^22^. The 2D psoriasis model resembled this AMP induction as the gene expression of *S100A7* (encodes psoriasin), *DEFB4A* (encodes hBD2) and *DEFB103A* (encodes hBD3) were induced. Stimulation with the plant extract mixture reduced the strong AMP induction to normal levels (Fig. 3a, c, e). Exemplified by *S100A7* and *DEFB4A*, it was demonstrated by ELISA that the extract lowered the corresponding protein expression levels of psoriasin and hBD2 in the 2D psoriasis model as well (Fig. 3b + d).

### Both *NFKBIZ* mRNA and IκBζ protein levels are induced in the 2D psoriasis model and inhibited by the plant extract mixture

**Fig. 4:**
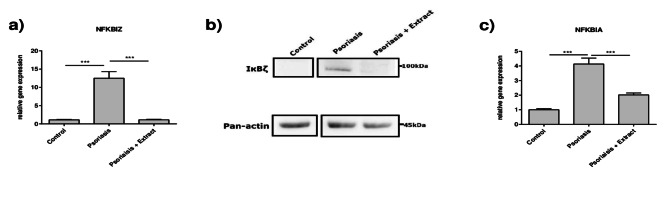
Plant extract mixture reduces *NFKBIZ* and *NFKBIA* gene expression and IκBζ protein levels. CaCl_2_-differentiated NHEKs were stimulated as described in Fig. 1. Gene expression levels of (**a**) *NFKBIZ* and (**c**) *NFKBIA* were measured and statistical significance was tested by one-way ANOVA with subsequent Sidak’s multiple comparisons test (*n* = 12; *p < 0.05; **p < 0.01; ***p < 0.001; ns = not significant). (**b**) Protein expression levels of IκBζ were assessed by western blot using an IκBζ antibody. Detection of pan-actin serves as a loading control. Uncropped blots are shown in Supplementary Figure S1.

It is known that the atypical nuclear NF-kappa-B inhibitor zeta (IκBζ), encoded by *NFKBIZ*, is a key regulator in psoriasis by mediating the induction of a selective subset of NF-κB target genes such as the measured *IL17C*, *IL36G*, *CSF2*, *CXCL8* and *DEFB4A*^23,24^. Therefore, *NFKBIZ* was analyzed to investigate whether the observed effects of the extract may be mediated via modulation of *NFKBIZ*. It was shown that the gene expression of *NFKBIZ* was induced in the 2D psoriasis model and the stimulation with the extract strongly reduced the expression to normal levels (Fig. 4a). Protein expression analysis of IκBζ by western blot confirmed the upregulation of IκBζ in the psoriasis model and its downregulation following treatment with the plant extract mixture (Fig. 4b). As *NFKBIZ* transcription is known to be primarily regulated via the NF-κB pathway^25^, it was further investigated, whether NF-κB is activated in the 2D psoriasis model and if the extract may exert its inhibitory effects through the inhibition of NF-κB signaling. Therefore, the gene expression level of *NFKBIA*, a direct target gene of the canonical NF-κB pathway^26^, was measured (Fig. 4c). *NFKBIA* gene expression was induced in the 2D psoriasis model and reduced by simultaneous incubation with the plant extract mixture, so it was hypothesized that the extract may inhibit NF-κB activity.

### Plant extract mixture activates the aryl hydrocarbon receptor in the 2D psoriasis model

**Fig. 5:**
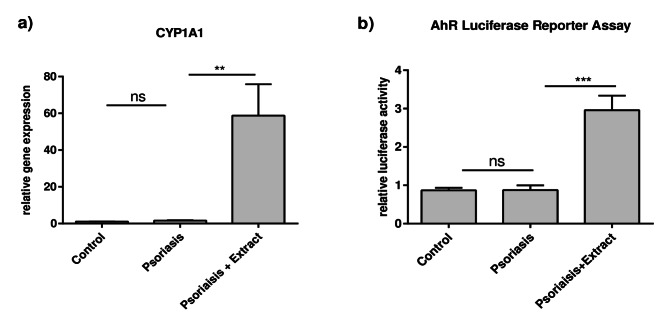
Plant extract mixture activates the AhR in the 2D psoriasis model. (**a**) CaCl_2_-differentiated NHEKs were stimulated as described in Fig. 1. Gene expression levels of *CYP1A1* were measured and statistical significance was tested by Kruskal-Wallis test with subsequent Dunn’s multiple comparisons test (*n* = 12). (**b**) To determine AhR activation, NHEKs were transfected with the pGudLuc6.1 plasmid containing *firefly* luciferase which expression depends on AhR activation and the pGL4.74 [*hRLuc*/TK] reference plasmid containing *renilla* luciferase. One day after transfection, cells were stimulated as described in (**a**). After cell lysis, activation of AhR was determined by measurement of relative luciferase activities. Statistical significance was tested by one-way ANOVA with subsequent Sidak’s multiple comparison test (*n* = 12; **p < 0.01; ***p < 0.001; ns = not significant).

In addition to the potential inhibition of NF-κB signaling, other pathways may be involved in the observed effects of the plant extract mixture, especially in regard to the skin barrier-inducing effects. Indeed, as previously reported, stimulation with the plant extract mixture led to the activation of the aryl hydrocarbon receptor (AhR) in a 2D and 3D atopic dermatitis-like skin model^21^. The AhR is an important factor in skin barrier regulation and activation of AhR by specific ligands has been shown to induce the expression of the barrier molecule *FLG* in keratinocytes^17^. Thus, we measured the gene expression of cytochrome P450 Family 1 Subfamily A Member 1 (*CYP1A1)*, a direct downstream marker of AhR activation, to determine if the AhR is also activated in the 2D psoriasis model. As shown in Fig. 5a, the gene expression of *CYP1A1* is induced by the extract, indicating AhR activation. Additionally, an AhR luciferase reporter assay confirmed the AhR activation (Fig. 5b). Of note, the psoriasis-cytokine stimulation alone seems to have no impact on *CYP1A1* gene expression or the AhR activation in comparison to untreated cells.

### AhR mediates the *FLG*-inducing effects of the plant extract mixture

**Fig. 6:**
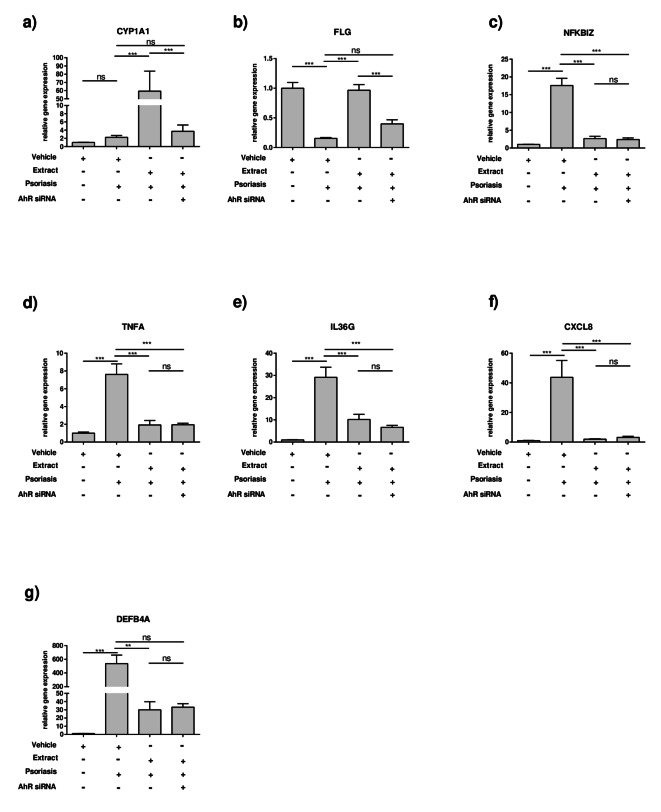
Downregulation of AhR inhibits the filaggrin-inducing effects but not the anti-inflammatory effects of the plant extract in the 2D psoriasis model. NHEKs were transfected with control or AhR siRNA and stimulated as described in Fig. 1. Gene expression levels of (**a**) *CYP1A1*, (**b**)* FLG*, (**c**)* NFKBIZ*, (**d**)* TNFA*, (**e**) *IL36G* (**f**)* CXCL8* and (**g**)* DEFB4A* were measured. Statistical significance was determined by (**a**–**f**) one-way ANOVA with subsequent Sidak’s multiple comparison test or (**c**) Kruskal-Wallis with subsequent Dunn’s multiple comparison test (n = 9; *p< 0.05; **p < 0.01; ***p 0.001; ns = not significant).

Next, the AhR was inhibited by AhR siRNA to determine which effects by the extract may be due to the activation of AhR (Fig. 6). As a result, the upregulation of *CYP1A1* by the extract was inhibited by AhR siRNA (Fig. 6a). Additionally, the induction of *FLG* gene expression was also lowered by AhR inhibition (Fig. 6b). But the AhR suppression via siRNA had no impact on the downregulation of inflammatory mediators such as *NFKBIZ*,* TNFA*, *IL36G* and *CXCL8* (Fig. 6c-f) or of the *DEFB4A* gene expression (Fig. 6g) by the extract.

### Plant extract mixture exhibits antioxidant effects in the 2D psoriasis model

**Fig. 7:**
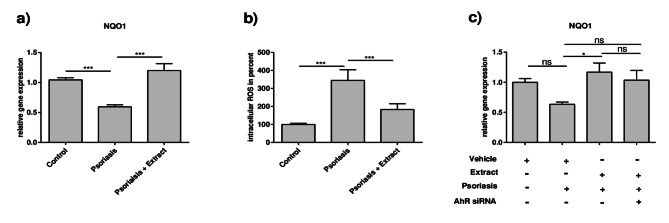
The plant extract mixture exhibits antioxidant effects in the 2D psoriasis model. CaCl_2_-differentiated NHEKs were stimulated as described in Fig. 1. (**a**) Gene expression levels of *NQO1* were measured and statistical significance was tested by one-way ANOVA with subsequent Sidak’s multiple comparisons test (*n* = 12). (**b**) Intracellular reactive oxygen species were measured by DCFDA-based assay. The mean fluorescence value of the control cells was set to 100%, and the relative intracellular ROS levels of the other samples were calculated as percentages of the control. Statistical significance was tested by Kruskal-Wallis test with subsequent Dunn’s multiple comparison test (*n* = 12, *p < 0.05; **p < 0.01; ***p < 0.001; ns = not significant). (**c**) NHEKs were transfected and stimulated as described in Fig. 6. Gene expression levels of *NQO1* were measured and statistical significance was determined by one-way ANOVA with subsequent Sidak’s multiple comparison test (*n* = 9; *p < 0.05; ns = not significant).

Besides skin barrier-inducing effects, it was reported that specific AhR activators exhibit antioxidant effects^27^. Because oxidative stress is a factor involved in the pathogenesis of psoriasis^8^, we determined if the plant extract mixture leads to an antioxidant response in keratinocytes. Therefore, we analyzed the gene expression of the antioxidant enzyme *NQO1*, which was lowered in the 2D psoriasis model. Adding the plant extract mixture to this model restored this downregulation again (Fig. 7a). Furthermore, measurement of intracellular reactive oxygen species (ROS) in the 2D psoriasis model via 2′,7′-dichlorofluorescin diacetate (DCFDA)-based assay exhibited an elevated ROS level in the 2D psoriasis model, which was reduced by simultaneous stimulation with the plant extract (Fig. 7b). Interestingly, the *NQO1* induction by the plant extract in the 2D psoriasis model was not affected by AhR knockdown via siRNA (Fig. 7c).

## Discussion

Based on the findings of our previous study, which demonstrated that a plant extract mixture activating the AhR has beneficial effects in an atopic dermatitis (AD) in vitro model^21^, the present study aimed to investigate the effects of the same extract in the context of psoriasis. The decision for extending the investigation of the plant extract mixture to psoriasis was based on the fact that other AhR agonists such as tapinarof exhibit both positive effects on AD and psoriasis^19^. In line with that, our current findings demonstrate that the plant extract mixture has beneficial effects in the 2D psoriasis model as well. This was demonstrated by anti-inflammatory effects, antioxidant effects and barrier-strengthening effects. The combination of anti-inflammatory activity and skin barrier restoration may be particularly promising for the treatment of psoriasis, as it addresses both the hallmark inflammation and the defective epidermal barrier in psoriatic lesions^28^. Besides the psoriasis-typical strong hyperproliferation of keratinocytes, aberrant differentiation contributes directly to the disruption of the epidermal barrier^5^. A pivotal factor in the development of a functional epidermal barrier is the cornified envelope protein filaggrin (FLG)^29^. In psoriasis patients, it was observed that the expression of *FLG* is reduced in lesional and even in non-lesional skin^30^. Consistently, stimulation with the pro-inflammatory cytokines IL-17A, IL-22, IL-1β and TNFα inhibited the gene expression of *FLG* in our 2D psoriasis model. Interestingly, we found that cytokine treatment did not alter involucrin gene expression in the 2D psoriasis model and the stimulation with the plant extract mixture did not change the gene expression of involucrin. This is in contrast to the observed gene expression of the late differentiation marker filaggrin and loricrin, which were both downregulated by the psoriasis-like cytokine treatment and induced by the plant extract mixture. These differences are consistent with previous studies reporting that involucrin is regulated differently from filaggrin and loricrin in psoriatic environment. For example, TNFα reduces the expression of filaggrin and loricrin^30^, but upregulates involucrin expression in keratinocytes^31^. Concordantly, TNFα antibody treatment in psoriasis patient induces filaggrin and loricrin, but has no effect on involucrin^30^. Based on our cytokine mixture consisting of IL-22, IL-17A, TNFα, and IL-1β, we did not expect a reduction in involucrin gene expression in our 2D psoriasis model. Rather, we anticipated an induction, as it is known that TNFα, IL-1 and IL-17A upregulate involucrin^31,32^. However, IL-22 downregulates involucrin^33^ and may therefore counteract the inducing effects of the other cytokines, resulting in unchanged involucrin gene expression in the 2D psoriasis model.

As mentioned above, the downregulation of *FLG* was restored by the plant extract. AhR suppression by siRNA abolished this restoration of *FLG* expression, demonstrating that AhR activation by the plant extract mixture was essential for the *FLG*-inducing effect. The induction of barrier genes in human keratinocytes by various AhR activators was reported before^18,34,35^. Thus, AhR activation may be beneficial in skin diseases with a disturbed barrier function. Accordingly, many studies have focused on the impact of AhR activation in the context of atopic dermatitis, in which a dysregulated skin barrier is considered a central hallmark^36–39^. It was also shown that inactivation of AhR activity suppresses epidermal differentiation in human keratinocytes, revealing the AhR as an important factor for physiological keratinocyte differentiation^36^. Since psoriasis is associated with an impaired keratinocyte differentiation process, it is likely that disturbed AhR signaling may contribute to the skin barrier defect observed in psoriasis. However, investigations of AhR activity in psoriatic human skin are limited and the results are not consistent. Many studies reported a higher expression of AhR in lesional skin of psoriasis patients compared with healthy skin, but some of them measured an increase of its downstream marker CYP1A1 while others determined less CYP1A1 expression^40,41^. Furthermore, Kyoreva and colleagues observed lower expression of CYP1A1 in skin biopsies of lesional psoriatic skin, but measured a higher CYP1A1 activity in human primary blood cells^42^. Together, despite these inconsistencies, a dysregulation of AhR seems to be likely. An exact clarification of the AhR regulation in context of psoriasis remains challenging due to its multifactorial modulation, combined with cell type-specific effects^43^. Studies with larger sample sizes and analysis of specific cell populations may help to clarify the role of AhR signaling in psoriatic skin.

Besides filaggrin-inducing effects, AhR activation by specific ligands is also connected with anti-inflammatory effects in psoriasis^18,20^. In an imiquimod (IMQ)-induced psoriasis mouse model, topical treatment with the endogenous AhR ligand 6-formylindolo[3,2-b] carbazole (FICZ) or tapinarof led to a reduced expression of proinflammatory cytokines such as IL-17A and IL-17F. This effect could not be observed in AhR knockout mice, confirming the crucial role of AhR for the observed anti-inflammatory effect^18^. In line with this, di Meglio and colleagues demonstrated that FICZ reduces the expression of inflammatory genes in human psoriatic skin biopsies, whereas the AhR antagonist CH-223,191 enhances them. AhR-deficiency in an IMQ-induced mouse model results in a more severe inflammation, while FICZ treatment again has anti-inflammatory effects^20^. Bone-marrow chimera experiments revealed that the enhanced inflammation was only achieved when the AhR was absent in non-hematopoietic skin cells, including keratinocytes, demonstrating the important role of AhR in these cells for regulating inflammation^20^.

In our current study, the plant extract mixture exhibited an anti-inflammatory effect in the 2D psoriasis model. For example, we observed induction of the genes encoding the proinflammatory IL-1 cytokines IL-1β and IL-1α. The same pattern was observed for the IL-1 receptor antagonist IL-1ra (*IL1RN*), which interferes with the IL-1 receptor and thereby block IL-1α- and IL-1β-mediated signal transduction^44^. This observation supports the model in which IL-1α or IL-1β drive *IL1RN* expression, resulting in a negative feedback loop that limits IL-1 signaling^44^. In addition to IL‑1, it has been reported that other cytokines included in our psoriasis-like cytokine mixture, such as IL‑17 and TNF‑α, also induce IL1RN expression^45^. Elevated levels of IL1RN were determined in skin of psoriasis patients as well^46^, which may represent a compensatory mechanism to attenuate IL-1–driven inflammation.

Surprisingly, the observed anti-inflammatory effects seem to be AhR-independent as AhR suppression via siRNA did not attenuate the reduction in inflammatory gene expression. While the AhR activation seems not to be involved in the anti-inflammatory effects in keratinocytes, future studies may examine whether the plant extract mixture might exert AhR-dependent anti-inflammatory effects in other cell types such as immune cells. Indeed, AhR is known to play an important role for Th17/22 and regulatory T cell (Treg) differentiation^19^. For example, the anti-inflammatory effects of tapinarof observed in vivo may be partly mediated by a suppression of Th17 response, as demonstrated in stimulated CD4 + cells and in an ex vivo skin T-cell assay^18^. However, it seems to be ligand-, time- and dose-dependent, whether activation of AhR promotes or suppresses Th17 or Treg differentiation^47,48^. Thus, as psoriasis is also considered a T-cell-driven disease, it would be interesting to investigate the influence of the plant extract on T cells in future studies.

As the anti-inflammatory effect observed in keratinocytes is AhR-independent, the extract may modulate other psoriasis-related inflammatory signaling pathways in these cells. The transcription factor nuclear factor kappa B (NF-κB) is activated in inflammatory environments and plays a crucial role in psoriasis by regulating the expression of different psoriasis-relevant genes^49^. Importantly, the atypical IκB member IκBζ (encoded by *NFKBIZ)* is required as mediator for the regulation of many of these proinflammatory molecules and antimicrobial peptides^50^. In particular, IκBζ has been identified as a crucial mediator of IL-17A-induced gene expression^24,51,52^. Furthermore, *NFKBIZ* is upregulated in psoriatic skin and IMQ-treated *Nfkbiz*-deficient mice do not show skin inflammation, highlighting *NFKBIZ* as a key player in psoriasis pathogenesis^24^. Interestingly, keratinocyte-derived IκBζ seems to play an important role for the induction of psoriatic inflammation and skin lesions^53^. Thus, we determined the effects of the plant extract mixture on the NF-κB/IκBζ pathway in the 2D psoriasis model by measuring the gene expression of *NFKBIZ* and *NFKBIA*, a direct NF-κB downstream target^54^. As expected, both genes were induced in the 2D psoriasis model. In line with that, protein levels of IκBζ were induced as well. Stimulation with the plant extract mixture inhibited the expression of *NFKBIA* as well as *NFKBIZ* and its protein product, IκBζ, suggesting that the NF-κB pathway may be suppressed by the plant extract. Especially the suppression of *NFKBIZ/*IκBζ may be responsible for the plant extract-mediated decrease in inflammatory genes and AMP expression in our 2D psoriasis model. Nevertheless, further investigations are required to validate this hypothesis. In addition, the potential impact of the plant extract mixture on other psoriasis-relevant pathways, such as MAPK, should also be considered.

Furthermore, there is increasing evidence that oxidative stress also plays an important role in psoriasis pathogenesis^8^. Different studies reported elevated levels of reactive oxygen species and a dysregulated antioxidative response in samples of psoriasis patients^8,9^. The resulting oxidative stress can directly contribute to inflammation by damaging cellular biomolecules^55^, and indirectly by modulating different redox-sensitive key pathways in psoriasis^56^. For example, TNFα-induced cytokine expression in keratinocytes is partly mediated via ROS generation and suppressed by quenching of ROS^57^. Similarly, IL-17A-induced psoriasis-associated metabolic changes in keratinocytes are reversed by blocking ROS^58^. Additionally, the expression of the proangiogenic factor VEGF is induced by ROS^59^, and some antioxidant compounds reduce the expression again^60^. Targeting oxidative stress may therefore have positive effects on psoriasis. In our 2D psoriasis model, the psoriasis-typical cytokines induced an elevated level of ROS in keratinocytes, confirming the association between inflammation and oxidative stress. Treatment of the 2D psoriasis model with the plant extract reduced the amount of intracellular reactive oxygen species again. This reduction may be caused by direct radical scavenger effects of the plant extract, mediated by secondary plant metabolites such as polyphenols^61^. Indeed, we previously determined that the plant extract had a high radical scavenging activity against the 2,2-Diphenyl-1- picrylhydrazyl (DPPH) radical^62^. Beyond that, the antioxidant effect may be mediated by activation of antioxidant signaling pathways in keratinocytes, as many other AhR activators exhibit antioxidant effects by modulating the antioxidant Nrf2 pathway^18,36,37,63^. Concordantly, we detected an induction of *NQO1*, an antioxidant enzyme and downstream target of Nrf2 signaling, by the plant extract in the 2D psoriasis model. But this induction seems to be AhR-independent as AhR suppression by siRNA did not impact the *NQO1* induction. Previously, we also detected an AhR-independent induction of *NQO1* in an in vitro atopic dermatitis model^21^. Further experiments are required to verify whether the plant extract activates the Nrf2 pathway and to clarify the underlying mechanisms. In general, the exact role of Nrf2 in psoriasis pathogenesis is still under debate and both elevated and decreased levels of Nrf2 were observed in psoriatic skin^64,65^. Furthermore, Nrf2 activation may have beneficial effect on inflammation in psoriatic skin by reducing oxidative stress^66^, but on the other hand, Nrf2 activation may also have detrimental effects by promoting proliferation of keratinocytes^64^. Thus, when targeting the Nrf2 pathway in psoriasis, the potential proliferative effects on keratinocytes should be carefully considered^67^.

In summary, the results of our study demonstrated that the plant extract mixture under investigation exhibits anti-inflammatory, antioxidant and barrier-strengthening effects in a 2D psoriasis model. Thus, this plant extract mixture may offer a potential topical therapeutic option to treat psoriasis. This promising hypothesis has to be verified in more complex skin models and by in vivo studies. Because our study focused on investigating the plant extract’s effects in the context of psoriasis, a complex and time-consuming chemical characterization of the extract was not performed, as this was beyond the scope of the study. However, a future analysis of the chemical characteristics of the plant extract mixture would provide important insights and may be useful to improve the mechanistic understandings.

## Materials and methods

### Plant extract preparation

The same plant extract mixture was used, as described before^21^. In short, vacuum-dried apple extract (Herbstreith & Fox KG, Neuenbürg/Württ, Germany), curly kale extract (Anklam Extract GmbH, Anklam, Germany) and green tea extract (Eurochem Feinchemie GmbH, Gröbenzell, Germany) were solved together in 50% EtOH to an initial concentration of 100 mg/mL of each extract. 50% EtOH was chosen due to the complete solubility of the extract and because hydroethanolic extracts have been reported to contain a high amount of phenolic and flavonoid compounds^68,69^. This extract mixture was further diluted 1:10 in cell culture medium (DermaLife Basal medium supplemented with 6mM L-glutamine, CellSystems, Troisdorf, Germany) to a stock solution of 5% EtOH in cell culture medium containing 10 mg/mL of each single extract. For stimulation experiments, cells were treated with a 1:800 dilution of this plant extract mixture stock solution or with the corresponding adequate dilution of 5% EtOH as vehicle control. A single extract dose of 1:800 was chosen based on preliminary experiments identifying a biologically effective and non-toxic concentration. In detail, NHEKs stimulated with different concentration of the plant extract mixture revealed morphological changes with the extract at 1:400 dilution. A resazurin viability assay revealed reduced cell viability at 1:400 dilution indicating cytotoxic effects, whereas no changes in cell viability was observed at 1:800 dilution or lower (Supplementary Fig. 2). However, extracts at concentrations lower than 1:800 had fewer effects in the cell stimulation experiments (data not shown). In addition, a 1:800 dilution allows a direct comparison with the effects reported previously in the atopic dermatitis 2D model^21^. Thus, we used the plant extract at a 1:800 dilution for subsequent experiments.

### Culture and stimulation of primary normal human epidermal keratinocytes

For the 2D psoriasis model, primary normal human epidermal keratinocytes (NHEKs, pooled from four donors, PromoCell, Heidelberg Germany), used at passage 4, were seeded in a 24-well plate in Derma Life Complete medium (CellSystems, Troisdorf, Germany). When reaching 90–100% confluency, differentiation was induced by incubating the cells in the cell culture medium containing 1.3 mM CaCl_2_ for 48 h. Differentiated NHEKs were left untreated (Control) or treated with a cytokine cocktail (IL-22, IL-1β, TNFα and IL-17A (10 ng/ml each)) to simulate psoriasis-like inflammation. To determine the effects of the plant extract mixture on this 2D psoriasis model, the cytokine-treated cells were in parallel incubated with vehicle (5% EtOH) (Psoriasis) or the plant extract mixture (Psoriasis + Extract), both 1:800 diluted, for 21 h at 37 °C, in 5% CO_2_ atmosphere. Then, the supernatant was harvested for subsequent protein determination via ELISA. Cells were washed with phosphate-buffered saline (PBS, Thermo Fisher Scientific, Dreieich, Germany) and used for RNA isolation.

### RNA isolation and gene expression analysis by quantitative real-Time PCR

For determination of the gene expression levels of different genes, RNA was isolated, transcribed to cDNA and measured via quantitative real-time PCR as described before^21^. The gene expression levels of different psoriasis-related genes were measured and the respective intron-spanning primers are listed in Table 1. All gene expression levels were normalized to the constitutively expressed ribosomal protein L38 (*RPL38*) gene.

**Table 1:** List of intron-spanning primers used for gene expression analysis.

Gene	Forward Primer	Reverse primer
Colony Stimulating Factor 2,* CSF2*	5‘-CAGAAATGTTTGACCTCCAGGAGC-3‘	5‘-CTGGCCATCATGGTCAAGGG-3‘
Cytochrome P450 Family 1 Subfamily A Member 1, *CYP1A1*	5’- CACCATCCCCCACAGCAC-3’	5’-ACAAAGACACAACGCCCCTT-3’
CXC-motif chemokine ligand 8,* CXCL8*	5’-TCCTGATTTCTGCAGCTCTGT-3’	5’-AAATTTGGGGTGGAAAGGTT-3’
Defensin Beta 103B,* DEFB103B*	5’-TGTTTGCTTTGCTCTTCCTGT-3’	5’-CGCCTCTGACTCTGCAATAA-3’
Defensin Beta 4 A,* DEFB4A*	5´- GCCTCTTCCAGGTGTTTTTG − 3´	5´-GAGACCACAGGTGCCAATTT − 3´
Filaggrin,* FLG*	5′-GGCAAATCCTGAAGAATCCAGATG-3′	5′-GGTAAATTCTCTTTTCTGGTAGACTC-3’
Interleukin 1 Alpha,* IL1A*	5’-TGTGACTGCCCAAGATGAAG-3’	5’-AAGTTTGGATGGGCAACTGA-3’
Interleukin 1 Receptor Antagonist, *IL1RN*	5’-ATGGAGGGAAGATGTGCCTG-3’	5’-GTCCTGCTTTCTGTTCTCGC-3’
Interleukin 1 Beta,* IL1B*	5‘-AAGCCCTTGCTGTAGTGGTG-3‘	5‘-GAAGCTGATGGCCCTAAACA-3‘
Interleukin 17 C,* IL17C*	5‘-CTCAGCTACGACCCAGTGC-3‘	5‘-AAGGCCAGCTTCTGTGGATAG-3‘
Interleukin 36 Gamma,* IL36G*	5’-GTGTAAACCTATTACTGGGAC-3’	5’-TTGGAGGAGGCAATGAAC-3’
Involucrin,* IVL*	5’-CTGCCTCAGCCTTACTGTGA-3’	5’-GGAGGAGGAACAGTCTTGAGG-3’
Loricrin,* LOR*	5‘-CTCTCCTCACTCACCCTTCCT-3‘	5‘-AGGTCTTCACGCAGTCCAC-3‘
NFKB Inhibitor Alpha,* NFKBIA*	5‘-GTCAAGGAGCTGCAGGAGAT-3‘	5‘-TCATGGATGATGGCCAAGT-3‘
NFKB Inhibitor Zeta,* NFKBIZ*	5‘-ACACCCACAAACCAACTCTGG-3‘	5‘-TGCTGAACACTGGAGGAAGTC-3‘
NAD(P)H Quinone Dehydrogenase 1,* NQO1*	5’-AGCTCACCGAGAGCCTAGTT-3	5’-GTGCTCTTCTGCCGACCAT-3’
Homo sapiens ribosomal protein L38, *RPL38*	5′-TCAAGGACTTCCTGCTCACA-3′	5′-AAAGGTATCTGCTGCATCGAA-3′
S100 Calcium Binding Protein A7,* S100A7*	5‘-GATTGAGAAGCCAAGCCTGC-3‘	5‘-GGCGAGGTAATTTGTGCCCT-3‘
Tumor necrosis factor alpha,* TNFA*	5’-CCTGCTGCACTTTGGAGTG-3’	5’-GCTTGAGGGTTTGCTACAACA-3’
Vascular Endothelial Growth Factor A,* VEGFA*	5‘-CTACCTCCACCATGCCAAGT-3‘	5‘-AGCTGCGCTGATAGACATCC-3‘

### Detection of protein concentration via ELISA

The protein concentration of hBD2, psoriasin, TNFα and IL-8 in the cell supernatants was evaluated by enzyme-linked immunosorbent assay (ELISA). Quantification of hBD2 was done by hBD2 ELISA, as previously described^70^ with some modifications: For the washing steps, PBS + 0.05% TWEEN 20 (Sigma-Aldrich Chemie GmbH, Taufkirchen, Germany) was used instead of PBS + 0.1% TWEEN 20. The blocking time and the incubation with the samples and standard curve was increased to 1 h at 37 °C. Furthermore, TMB High Sensitivity Substrate Solution (Biolegend GmbH, Koblenz, Germany) instead of ABTS was used as development agent and thus, the absorbance was measured at 600 nm in a microplate reader (Multiscan Sky, Thermo Scientific, Dreieich, Germany). Psoriasin levels were analyzed by psoriasin ELISA according to a standard protocol^71^. TNFα and IL-8 concentrations were determined using commercial ELISA kits (human TNFα or human CXCL8/IL-8 DuoSet ELISA, R&D Systems, Minneapolis, MN, USA) following the manufacturer’s protocol.

### Western blot analysis

NHEKs were washed with PBS and lysed by scraping them directly from the culture dish in lysis buffer (10 mM Tris pH 8, 1 mM EDTA, 0.5 mM EGTA, 1% Triton-X 100, 0.1% sodium deoxycholate, 0.1% SDS, 140mM NaCl and 1x protease inhibitor cocktail (Roche, Basel, Switzerland)). Cell lysates were then briefly sonicated (UP200St Hielscher, Hielscher Ultrasonic, Teltow, Germany) and centrifuged at 12,000 *g* for 3 min. Supernatants were loaded on an SDS page (10% NuPAGE Bis-Tris gel, Invitrogen, Carlsbad, CA, USA) for protein separation and then transferred to a polyvinylidene difluoride (PVDF) membrane. IκBζ was detected by using an anti–IκBζ antibody (1:500 diluted, Cell Signaling Technology, cat. no. 9244, Danvers, MA, USA), followed by incubation with an HRP-conjugated goat anti-rabbit IgG secondary antibody (1:10,000 diluted, Peroxidase AffiniPure^®^ Goat Anti-Rabbit IgG (H + L), Jackson ImmunoResearch, West Grove, PA, USA). To normalize protein levels, pan-actin was detected as reference protein by incubation with a pan-actin antibody (1:500 diluted, Cell Signaling Technology, cat. no. 4968, Danvers, MA, USA), followed by incubation with the HRP-conjugated goat-anti rabbit IgG secondary antibody (1:10,000 diluted, Peroxidase AffiniPure^®^ Goat Anti-Rabbit IgG (H + L), Jackson ImmunoResearch, West Grove, PA, USA). Signals were visualized using an ECL staining reagent (ECL Select^TM^Western Blotting Detection Reagent, Cytiva, Freiburg, Germany).

### Measurement of AhR activation via AhR luciferase activity assay

To determine the activity of the transcription factor AhR, NHEKs were transfected with the pGudLuc6.1 reporter plasmid (300ng/well, generously gifted by M. Denison, U.C. Davis) by using the transfection reagent FuGENE HD (Promega, Madison, WI, USA). In the reporter plasmid, the expression of *firefly* luciferase depends on AhR activation. Additionally, NHEKs were co-transfected with the pGL4.74 [*hRluc*/TK] *renilla* luciferase plasmid (Promega, Madison, WI, USA) (30 ng/well), which functioned as a reference plasmid to monitor transfection efficiency. Six hours after transfection, medium was changed. At 100% confluency, transfected cells were stimulated with the psoriasis-like cytokine cocktail together with the plant extract mixture or vehicle for 21 h as described in the in vitro stimulation experiments. Luciferase activities were measured according to the protocol of the *firefly*/*renilla* Dual Luciferase activity assay (Sigma-Aldrich Chemie GmbH, Taufkirchen, Germany). The ratio between *firefly* and *renilla* luciferase activities in each sample was determined as a measurement of AhR activation.

### AhR deficiency induced by siRNA

AhR-deficient NHEKs were generated by transfection with AhR siRNA as previously described^21^. Therefore, 1 µL HiPerfect transfection reagent (Qiagen, Hilden, Germany) was used to transfect 70% confluent NHEKs with 5 nM AhR siRNA (AHR Silencer^®^select siRNA S1199, Ambion) or non-silencing control siRNA (Silencer^®^select negative control siRNA, Ambion) per well. At 100% confluency, transfected NHEKs were used for stimulation experiments as described above.

### Determination of intracellular reactive oxygen species in NHEKs

Intracellular reactive oxygen species (ROS) in NHEKs were quantified by using the DCFDA Cellular ROS detection assay kit (Abcam, Cambridge, UK) as described previously^21^. In short, 100% confluent NHEKs were stimulated with the psoriasis-like cytokine cocktail and vehicle (Psoriasis) or the plant extract mixture (Psoriasis + Extract), both 1:800 diluted, for 21 h. NHEKs stimulated with vehicle only functioned as control. Subsequently, cells were washed with PBS and stained using the DCFDA assay as outlined in the manual. Fluorescence was determined in a plate reader (Infinite M Plex, Tecan, Crailsheim, Germany) with extinction wavelength of 485 nm and emission wavelengths of 535 nm. The background fluorescence, determined from unstained and untreated keratinocytes, was subtracted from the measured fluorescence values of each well. The mean fluorescence value of the control cells was then set to 100%, and the relative intracellular ROS levels of the other samples were calculated as percentages of the control.

### Statistics

Statistical analysis was performed by using GraphPad Prism 6.0 (GraphPad Software, San Diego, CA, USA). Data are presented as mean + standard error of mean (SEM). The Shapiro–Wilk test was used to assess normality. For multiple comparisons of normally distributed data, one-way ANOVA followed by Sidak’s multiple comparison test was used. The non-parametric Kruskal-Wallis test with subsequent Dunn’s multiple comparison test was performed for multiple comparisons of non-normally distributed data. A p-value less than 0.05 indicates a significant difference.

## Supplementary Information

Below is the link to the electronic supplementary material.

Supplementary Material 1

## Data Availability

The datasets generated during the current study are available from the corresponding author on reasonable request.
