# Supplementary material for: Plant extract mixture shows anti-inflammatory and barrier-strengthening effects and activates aryl hydrocarbon receptor in a 2D psoriasis model
Source: Sci Rep. 2026 Apr 28;16:13638. doi: 10.1038/s41598-026-50000-8 (PMC13125211; doi:10.1038/s41598-026-50000-8)
Supplement: Supplementary file 1 — Supplementary Material 1 [file 41598_2026_50000_MOESM1_ESM.pdf]

Supplementary Information for:

Plant extract mixture shows anti-inflammatory and barrier-strengthening effects and activates aryl hydrocarbon receptor in a 2D psoriasis model.

Authors: Nina Heinemann[1], Franziska Rademacher[1], Henning Vollert[2], Regine Gläser[1], Jürgen Harder[1*]

[1] Department of Dermatology, Kiel University, Kiel, Germany

[2] Bioactive Food GmbH, Bad Segeberg, Germany

This file includes:

- Supplementary Methods
- Supplementary Figures S1-S2

Supplementary Methods

Resazurin cell viability assay

Cell viability of NHEKs was determined using the fluorometric Cell Viability Kit based on a resazurin assay (Promocell, Heidelberg, Germany). In detail, $CaCl_2$-differentiated NHEKs, seeded in a 96-well plate, were treated with 1:400, 1:800 and 1:1600 dilution of the plant extract mixture for 24 h. Then, medium was replaced with 100 µL cell medium containing 10% resazurin. After 2 h, absorbance was measured at 570 nm and 600 nm using a microplate reader. The difference in absorbance between 570 nm and 600 nm was calculated and normalized to the control (cells incubated with medium only).

Supplementary Figures

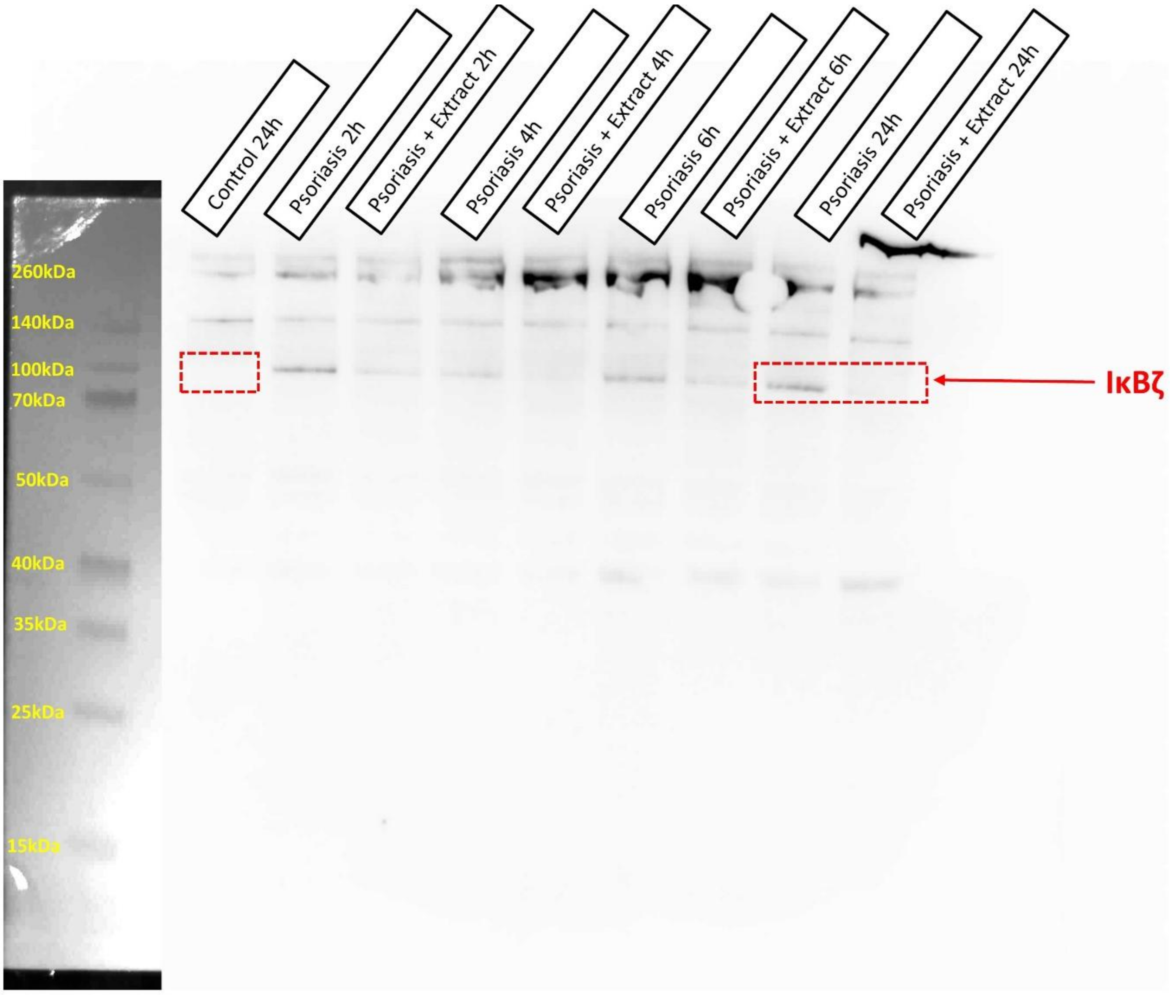

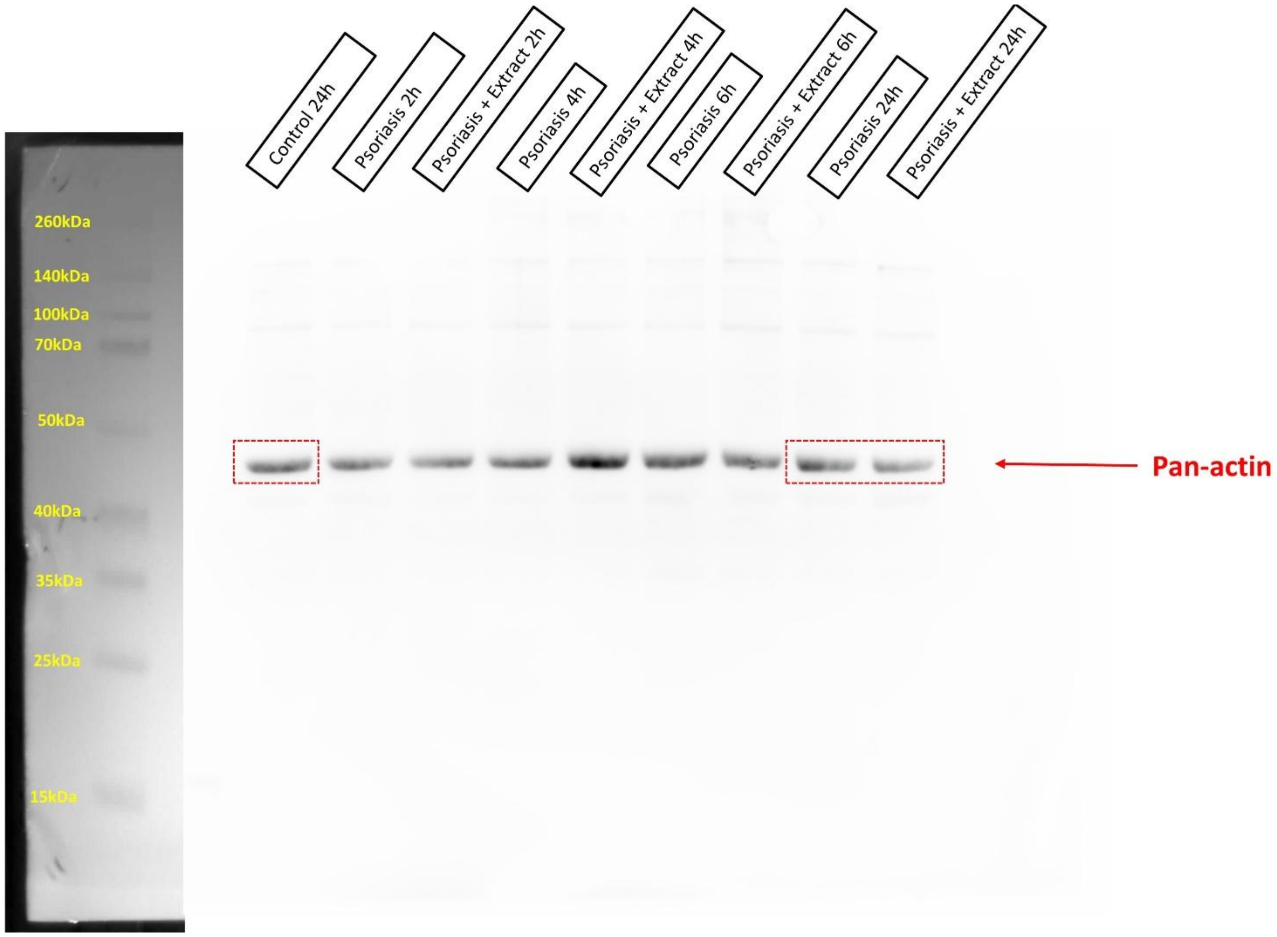

Supplementary Figure S1: Uncropped blots for IκBζ and pan-actin shown in Figure 4. Cropped areas used in the main figure are indicated by red boxes. The molecular weight markers are shown on the left of each blot.

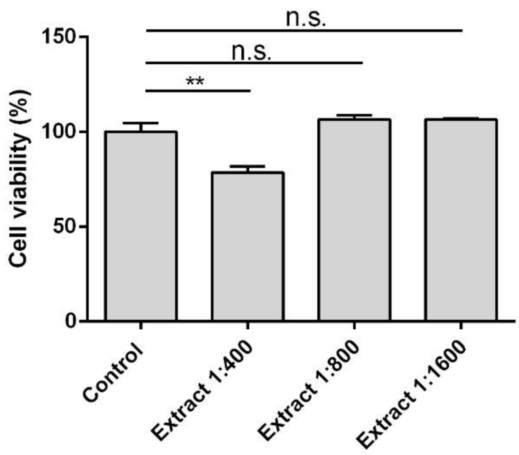

Supplementary Figure S2: Treatment of differentiated NHEKs with the plant extract mixture in 1:400 dilution reduced cell viability. $CaCl_2$-differentiated NHEKs were treated with different concentrations of the plant extract mixture for 24h. Then, cell viability was measured by using resazurin cell viability assay. Absorbance results were normalized to the control. Statistical analysis was tested by one-way ANOVA with subsequent comparison (n=3, **p < 0.01, ns = not significant). Bars indicate means + SEM.
